# The Swiss chiropractic practice-based research network: a population-based cross-sectional study to inform future musculoskeletal research

**DOI:** 10.1038/s41598-023-32437-3

**Published:** 2023-04-06

**Authors:** Rahim Lalji, Léonie Hofstetter, Alice Kongsted, Viktor von Wyl, Milo A. Puhan, Cesar A. Hincapié

**Affiliations:** 1 EBPI-UWZH Musculoskeletal Epidemiology Research Group, University of Zurich and Balgrist University Hospital, Forchstrasse 340, 8008 Zurich, Switzerland; 2 Epidemiology, Biostatistics and Prevention Institute (EBPI), University of Zurich, Zurich, Switzerland; 3 University Spine Centre Zurich (UWZH), Balgrist University Hospital, University of Zurich, Zurich, Switzerland; 4 Department of Sports Science and Clinical Biomechanics, University of Southern Denmark, Odense, Denmark; 5 Chiropractic Knowledge Hub, Odense, Denmark; 6 Institute for Implementation Science in Health Care, University of Zurich, Zurich, Switzerland

**Keywords:** Rehabilitation, Outcomes research, Health occupations, Epidemiology

## Abstract

The Swiss chiropractic practice-based research network (PBRN) is a nationwide project developed in collaboration with patients, clinicians, and academic stakeholders to advance musculoskeletal epidemiologic research. The aim of this study was to describe the clinician population recruited and representativeness of this PBRN to inform future collaboration. A population-based cross-sectional study was performed. PBRN clinician characteristics were described and factors related to motivation (operationalised as VAS score ≥ 70) to participate in a subsequent patient cohort pilot study were assessed. Among 326 eligible chiropractors, 152 enrolled in the PBRN (47% participation). The PBRN was representative of the larger Swiss chiropractic population with regards to age, language, and geographic distribution. Of those enrolled, 39% were motivated to participate in a nested patient cohort pilot study. Motivation was associated with age 40 years or older versus 39 years or younger (OR 2.3, 95% CI 1.0–5.2), and with a moderate clinic size (OR 2.4, 95% CI 1.1–5.7) or large clinic size (OR 2.8, 95% CI 1.0–7.8) versus solo practice. The Swiss chiropractic PBRN has enrolled almost half of all Swiss chiropractors and has potential to facilitate collaborative practice-based research to improve musculoskeletal health care quality.

**Trial registration**: Swiss chiropractic PBRN (ClinicalTrials.gov identifier: NCT05046249); Swiss chiropractic cohort (Swiss ChiCo) pilot study (ClinicalTrials.gov identifier: NCT05116020).

## Introduction

Musculoskeletal (MSK) pain conditions, such as neck pain and low back pain, are a leading cause of disability globally and are the most prevalent disease area which would benefit from rehabilitation^1^. One factor which may contribute to this disability burden is a lack of MSK health care quality^2,3^. Examples of substandard clinical management of MSK pain include an overutilization of diagnostic imaging, the over prescription of opioids, and the potential underutilization of nonpharmacological approaches^4–7^. As a large proportion of MSK pain is managed in primary care, efforts to improve the quality of care in these settings, such as the development of practice-based research networks (PBRNs), may play an important role in identifying, studying, and addressing similar practice-based gaps^8–10^.

PBRNs have been conceptualised as groups of at least 15 ambulatory practices or clinicians devoted to the care of patients and affiliated by a mission to investigate questions related to community-based practice^11^. The PBRN structure often transcends a single project, with participating clinicians/clinics engaged in research-related activities on an ongoing basis^11^. This form of participatory research offers distinct advantages for integrating research into practice and performing translational research^10,12^. For example, the Australian Chiropractic Research Network (ACORN) is a PBRN within the scope of chiropractic and MSK health^13,14^. Since launching in 2015 it has provided the necessary infrastructure to examine a range of questions related to chiropractic patient management^15–17^.

The overarching aim of this study is to describe the characteristics of chiropractors recruited to a newly developed Swiss chiropractic PBRN and the representativeness of the PBRN in comparison to the larger Swiss clinician population to facilitate subsequent collaborative practice-based research. The specific clinical objectives were to assess (1) clinician self-perceived confidence in the management of low back pain; and (2) biomedical versus biopsychosocial treatment orientation in the management of MSK conditions. Our feasibility objectives were to describe (1) the proportion of clinicians opting in for participation in the PBRN; and (2) the proportion of PBRN clinicians who would be motivated to participate in the first nested study (Swiss chiropractic cohort (ChiCo) pilot study) to be conducted through this newly developed PBRN. Motivated PBRN participants will be contacted first to aid in patient recruitment for the Swiss ChiCo pilot study. The Swiss ChiCo pilot study is registered as a 12-week prospective patient cohort pilot study to assess the feasibility of PBRN longitudinal data collection.

## Methods

### Study setting and design

The 2020–2025 strategy report of the Swiss Chiropractic Association (ChiroSuisse) outlines the development of a Swiss chiropractic PBRN as research priority^18^. Chiropractic in Switzerland is a government-recognized health profession (alongside medicine, dentistry, veterinary medicine, and pharmacology) which focuses on the management of MSK conditions through primarily manual care^19,20^. Approximately 98% of all chiropractors in Switzerland are members of ChiroSuisse (personal communication, April 22, 2021), which corresponded to 326 clinician members as of December 2021. Development of the Swiss chiropractic PBRN began in August 2020 through consultation with multiple stakeholder groups including ChiroSuisse, the Swiss Chiropractic Patient Association (Pro Chiropractic Switzerland), a small group of interested Swiss chiropractors, and an international group of MSK health researchers. To promote clinician and patient participant recruitment, our stakeholders outlined the importance of setting both clinical and feasibility primary aims and outcomes during initial project phases. We reported this population-based cross-sectional study according to the Strengthening the Reporting of Observational Studies in Epidemiology (STROBE for cross-sectional studies) statement (Supplemental Material 1)^21^.

### Ethics approval

The Swiss chiropractic PBRN was approved by the independent research ethics committee of Canton Zurich (BASEC-Nr: 2021-01479) and complies with international ethical standards as outlined by the Declaration of Helsinki.

### Study population and recruitment

All 326 registered active chiropractor members (fully licensed chiropractors and postgraduate assistant chiropractors) of ChiroSuisse were eligible and invited to participate in the PBRN. This included members with clinical practice locations in Switzerland and Liechtenstein. Further details of the study methods, including the patient and public involvement strategy are provided in the published study protocol^22^.

From September 9th, 2021 to December 19th, 2021, clinicians were provided the opportunity to sign up for the Swiss chiropractic PBRN through scanning a Quick Response (QR) code at an in-person ChiroSuisse event or through a web link via email invitation. Study information forms outlined duties of PBRN participation, namely a commitment to consider involvement and collaboration in ongoing self-selected research activities. Accordingly, clinician participants may be contacted to support future nested research projects, but at all times are able to choose their level of involvement. Only clinicians who completed the electronic informed consent and fully completed the entry questionnaire were considered as part of the PBRN and available for future nested study recruitment.

### Variables and outcome measures

All data was collected through a self-report electronic questionnaire using the Research Electronic Data Capture (REDCap) web application platform^23^. This questionnaire was modeled after other chiropractic and MSK-related PBRN entrance questionnaires, with adaptations made through consultation with study stakeholders when necessary^11^. Before full implementation, the PBRN entrance questionnaire was pilot tested by licenced chiropractors from all Swiss national language regions (German, French and Italian). During pilot testing, the questionnaire took approximately 10–12 min for a clinician to complete.

The PBRN questionnaire collected information on clinician demographics (sex, age, licensure status, self-reported practice years, primary language, clinic location), practice characteristics (number of chiropractors and other healthcare professionals within the same practice, self-reported recall of average number of patient visits and new patient visits seen per week over the last three months, frequency with which patient complaints are managed, frequency with which patient subgroups are managed), digitalization of chiropractic practices (use of an electronic health record (EHR) system, encrypted email use, and provision of virtual care or telehealth services) and how COVID-19 has affected clinical practice (change in clinician quality of life, change in patient numbers, and change in work hours). The variable clinical practice years was derived from the publicly available register of medical professions in Switzerland (MedReg)^24^.

Primary clinical outcome of perceived self-confidence for the management of low back pain was measured using the Practitioner Confidence Scale ((PCS) range 4–20, lower scores mean greater self-confidence^25^) and biomedical versus biopsychosocial treatment orientation was measured using the MSK version of the Pain Attitudes and Beliefs Scale (PABS-MSK, range 10–60 each, with higher scores meaning greater treatment orientation)^26^. The PABS-MSK consists of two scales, each scored separately representing either biopsychosocial or biomedical treatment orientation.

The primary feasibility outcome of motivation to participate in the nested Swiss ChiCo patient cohort pilot study was measured using a Visual Analog Scale ((VAS), range 0–100), higher scores indicate greater motivation). For this question, participants were asked “On a scale from 0–100 how motivated are you to participate in the patient cohort phase of the Swiss ChiCo pilot study”. A pragmatic decision was made a-priori to identify clinicians who reported a well above medium interest to participate in the Swiss ChiCo pilot study based on a VAS score of ≥ 70 (hereafter denoted as “motivated PBRN participants”)^22^. Only motivated PBRN participants were contacted for subsequent Swiss ChiCo pilot study recruitment. The PBRN entry questionnaire is provided in Supplemental Material 2.

### Statistical analysis

Data were extracted from REDCap into R (version 4.2.0) for analysis. Descriptive statistics were reported as raw numbers with percentages or means with standard deviations as appropriate. Primary clinical and feasibility outcomes were additionally described with 95% CIs for mean values and percentages. The study population was described by: (1) characteristics of all clinician members of ChiroSuisse (non-participants and PBRN participants combined); (2) characteristics of non-participants (non-participants of the PBRN—clinicians who did not respond to the PBRN request or explicitly chose not to participate); (3) characteristics of clinicians who consented to participate in the PBRN (PBRN participants only); (4) characteristics of clinicians who consented to participate in the PBRN and endorsed being motivated to participate in a subsequent Swiss ChiCo pilot study (motivated PBRN participants only). Motivated PBRN participants were a subset of the larger PBRN group. The distinction between PBRN participants and motivated PBRN participants was made to aid in clinician recruitment for the Swiss ChiCo pilot study and to assess the extent this profile was representative of the full PBRN.

Multivariable logistic regression was used to assess the association between clinician and practice characteristics (age, sex, practice size, language of practice, EHR use) and motivation to participate in the patient cohort pilot study (Yes/No, cut point operationalised as VAS score ≥ 70). Alpha level was set at 0.05 and results were reported as odds ratios (ORs) with 95% CIs. Independent variables included in the regression model were selected on the basis of clinical experience and prior analysis of factors related to EHR use for Swiss chiropractors^27^.

## Results

**Figure 1:**
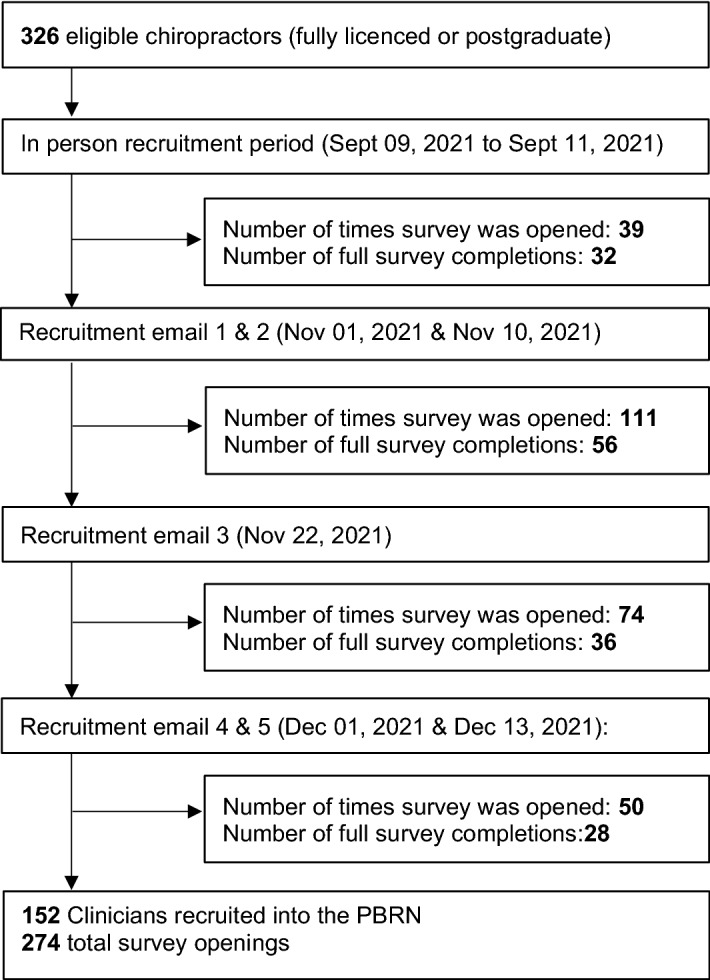
Swiss chiropractic PBRN recruitment flowchart. *Dec* December, *Nov* November, *PBRN* Practice-based research network, *Sept* September.

Of the 326 eligible chiropractors, 152 (46.6%) agreed to participate and completed the PBRN entrance questionnaire, 24 declined to participate and 150 did not respond (174 nonparticipants). Of those who declined, lack of time was reported as the most common reason for non-participation (50%). Further recruitment details are provided on Fig. 1**.**

### Clinician characteristics

Clinician participants of this Swiss chiropractic PBRN enrollment cross-sectional study were generally balanced in terms of sex (53% male versus 47% female) with an average age of 47 years (SD = 12.4). The majority were fully licenced chiropractors (87%), with the remainder being postgraduate chiropractors (13%) completing their clinical training in primary care settings across Switzerland. This translated to an average clinical practice experience of 19 years according to the Swiss MedReg register. The most common language participating clinicians use in their practice was reported as German (69%), followed by French (25%), Italian (5%) and finally Romansh (1%). Clinicians across a total of 71 different Swiss cities and 2 cities in Liechtenstein were represented in this PBRN study. The three cities with the largest number of participants were Zurich with 17 (11%), Bern with 10 (7%), and Biel-Bienne with 6 (4%). In total, 110 unique chiropractic clinics across Switzerland were represented within this PBRN study.

**Figure 2:**
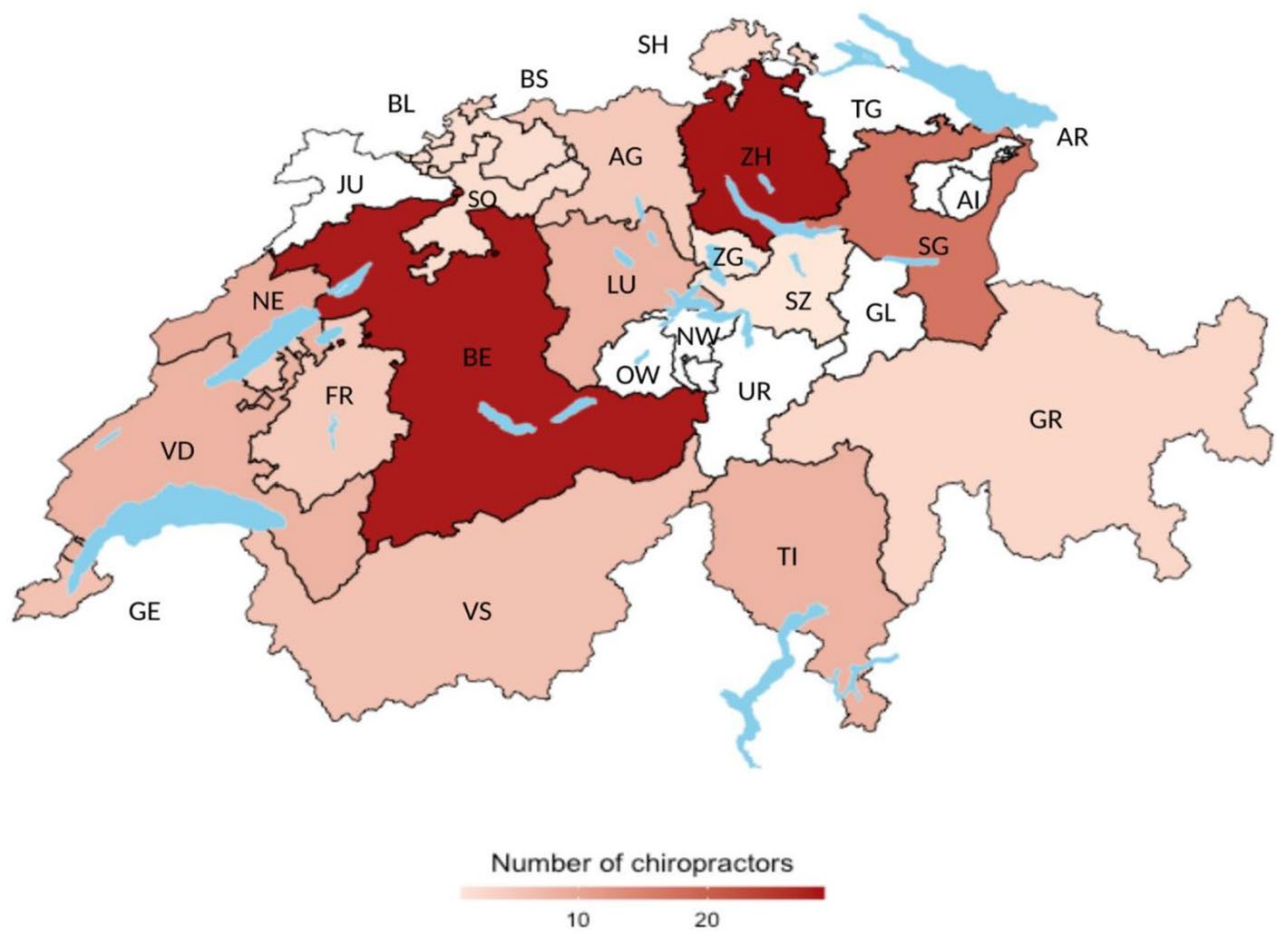
PBRN clinician participation across Swiss Cantons. *AG* Aargau, *AI* Appenzell Innerrhoden, *AR* Appenzell Ausserrhoden, *BE* Bern, *BL* Basel-Landschaft, *BS* Basel-Stadt, *FR* Fribourg, *GE* Genéve, *GL* Glarus, *GR* Graubünden, *JU* Jura, *LU* Luzern, *NE* Neuchâtel, *NW* Nidwalden, *OW* Obwalden, *SG* St. Gallen, *SH* Schaffhausen, *SO* Solothurn, *SZ* Schwyz, *TG* Thurgau, *TI* Ticino, *UR* Uri, *VD* Vaud, *VS* Valais, *ZG* Zug, *ZH* Zurich. Source of map: “RSwissMaps” package, R (Version 4.2.0, https://www.R-project.org/).

Table 1 provides an overview of clinician members of the Swiss chiropractic PBRN in relation to all clinician members of ChiroSuisse, non-participants, and clinician participants “motivated” to collaborate on the subsequent cohort pilot study (a subset of PBRN members). Members of the Swiss chiropractic PBRN were found to be broadly representative of the larger Swiss chiropractic community with regard to age (mean age: 47 vs 50 years), location of practice (Zurich: 11% vs 13%, Bern: 7% vs 6%, Biel/Bienne 4% vs 3%, St. Gallen 3% vs 2%) and primary language used in practice (German/Romansh: 70% vs 69%, French: 25% vs 27% and Italian: 5% vs 4%). The PBRN accounted for a greater amount of female participation (47% vs 35%) and a higher proportion of assistant/resident chiropractors compared to the larger Swiss chiropractic community. Figure 2 illustrates the geographical distribution of Swiss chiropractic PBRN participant clinicians across Switzerland.

**Table 1:** PBRN participating clinician characteristics in relation to all Swiss chiropractors and non-participants.

Characteristic	All Swiss chiropractors (n = 326)	Nonparticipants (n = 174)	Total PBRN participants (n = 152)	Motivated PBRN participants (n = 59)
Age group (years)—N (%)				
≤ 39	73 (22%)	26 (15%)	47 (31%)	15 (25%)
40–59	162 (50%)	81 (47%)	81 (53%)	35 (60%)
> 60	91 (28%)	67 (38%)	24 (16%)	9 (15%)
Mean age (SD)	50 (12.9)	54 (12.4)	47 (12.4)	46 (11.7)
Sex—N (%)				
Female	114 (35%)	43 (25%)	71 (47%)	31 (53%)
Male	212 (65%)	131 (75%)	81 (53%)	28 (47%)
Member status—N (%)				
Fully licenced	298 (91%)	166 (95%)	132 (87%)	53 (90%)
Assistant or resident	28 (9%)	8 (5%)	20 (13%)	6 (10%)
Years in practice—mean (SD)				
MedReg*	22 (12.6)	24 (11.8)	19 (12.8)	18.6 (11.0)
Location of practice—N (%)				
Zurich	41 (13%)	24 (14%)	17 (11%)	9 (15%)
Bern	21 (6%)	11 (6%)	10 (7%)	3 (5%)
Biel-Bienne	10 (3%)	4 (2%)	6 (4%)	5 (8%)
St. Gallen	8 (2%)	3 (2%)	5 (3%)	2 (3%)
Luzern	10 (3%)	5 (3%)	5 (3%)	1 (2%)
Geneva	16 (5%)	11 (6%)	5 (3%)	2 (3%)
La Chaux-de-Fonds	5 (2%)	0 (0%)	5 (3%)	2 (3%)
Basel	10 (3%)	6 (3%)	4 (3%)	0 (0%)
Wadenswil	4 (1%)	0 (0%)	4 (3%)	2 (3%)
Lausanne	11 (3%)	7 (4%)	4 (3%)	3 (5%)
Neuchatel	9 (3%)	4 (2%)	5 (3%)	1 (2%)
Bellinzona, Ascona, Lugano	7 (2%)	2 (1%)	5 (3%)	2 (3%)
Other	174 (53%)	97 (56%)	77 (51%)	27 (46%)
Primary language of practice—N (%)				
German/Romansh	225 (69%)	119 (68%)	106 (70%)	43 (73%)
French	89 (27%)	51 (29%)	38 (25%)	13 (22%)
Italian	12 (4%)	4 (2%)	8 (5%)	3 (5%)

*MedReg* register of medical professions, *n* number, *y* years.

### Clinical practice characteristics

A majority of participating chiropractors worked with other health care practitioners in intradisciplinary or interdisciplinary care settings. Sixty-five percent of participating chiropractors worked with other chiropractors within the same clinical setting, while 41% reported working with another type of health care professional. The most common health care professional within the same clinical settings, excluding another chiropractor, were massage therapists (71%), physiotherapists (44%) and medical physicians (29%). With regards to the number of patient visits, 39% of participants selected “50–99” for number of patients seen per week over the last 3 months and 44% selected the category of “7–12” for the average number of new patient visits per week over the last 3 months as the most often selected response for each question. Additional details regarding clinical practice characteristics are provided on Table 2**.**

**Table 2:** Practice characteristics of all PBRN clinics and motivated PBRN clinics.

Characteristic	PBRN participants (n = 152)	Motivated PBRN participants (n = 59)
Number of chiropractors within the practice—N (%)		
1	54 (35%)	15 (25%)
2 or 3	62 (41%)	27 (46%)
4 or more	36 (24%)	17 (29%)
Other healthcare professionals in practice—N (%)		
Yes	62 (41%)	20 (34%)
No	90 (59%)	39 (66%)
Number of other health care professionals in practice (participants, n = 62; motivated, n = 20)—N (%)		
1	18 (29%)	6 (30%)
2 or 3	17 (27%)	6 (30%)
4 or more	27 (44%)	8 (40%)
Type of health care professional within practice* (participants, n = 62; motivated, n = 20)—N (%)		
Physician	18 (29%)	7 (35%)
Physiotherapist	27 (44%)	9 (45%)
Massage therapist	44 (71%)	15 (75%)
Acupuncturist or nutritionist	9 (15%)	0 (0%)
Other	16 (26%)	2 (1%)
Patient visits per week per chiropractor—N (%)		
50 visits or less	12 (8%)	4 (7%)
50–99 visits	60 (39%)	21 (36%)
100–149 visits	42 (28%)	19 (32%)
150–199 visits	19 (13%)	6 (10%)
200–249 visits	10 (7%)	4 (7%)
More than 249 visits	9 (6%)	5 (8%)
New patient visits per week per chiropractor—N (%)		
0–6	62 (41%)	22 (37%)
7–12	68 (44%)	25 (42%)
13–20	15 (10%)	9 (15%)
More than 20 visits	7 (5%)	3 (5%)

*n* number.

*Participants allowed to select more than one response option.

### Primary clinical and feasibility objectives

Table 3 provides an overview of the primary clinical and feasibility outcomes. On average PBRN participating clinicians showed high confidence for managing patients with low back pain (5.6, SD = 1.8; 95% CI 5.3–5.9). The mean score on the biomedical subscale of the PABS-MSK was 32.5 (SD = 6.8; 95% CI 31.4–33.5), while mean score on the biopsychosocial subscale was 51.6 (SD = 5.0; 95% CI 50.9–52.5). 39% of participating clinicians were motivated (VAS score ≥ 70) to participate in the Swiss ChiCo pilot nested PBRN study (95% CI 30–46%) and average motivation to participate was 50.2 (SD = 32.3; 95% CI 45.0–55.6).

**Table 3:** Primary clinical and primary feasibility outcomes.

Outcome	PBRN participants	95% CI	Motivated PBRN participants	95% CI
Motivation to participate in patient cohort sub study				
Motivated to participate (VAS ≥ 70, score range, 0–100)—N (%)	59 (39%)	32–47%	–	–
Low motivation (VAS < 70, score range, 0–100)—N (%)	93 (61%)	53–68%	–	–
Average motivation (VAS, 0–100, score range, 0–100)—mean (SD)	50.2 (32.3)	45.0–55.6	84.3 (10.2)	81.7–87.0
Practitioner confidence in the management of LBP (PCS, score range 4–20)—mean (SD)	5.6 (1.8)	5.3–5.9	5.9 (1.9)	5.4–6.4
Pain attitudes and beliefs scale (PABS MSK)				
Biomedical Subscale (score range, 10–60)—mean (SD)	32.5 (6.8)	31.4–33.5	31.8 (6.5)	30.1–33.5
Biopsychosocial Subscale (score range, 10–60)—mean (SD)	51.6 (5.0)	50.9–52.5	52.7 (4.5)	51.4–53.8

*CI* confidence intervals, *PABS-MSK* pain attitudes and beliefs scale-musculoskeletal version, *PCS* practitioner self-confidence scale, *SD* standard deviation, *VAS* visual analog scale.

### Frequency of patient subgroups managed

Supplemental Material 3 outlines the frequency with which participating clinicians managed specific patient subgroups on a scale from “often” to “never”. Older persons (≥ 65 years) were reported as “often” managed within clinical practice by 89% of participants. A smaller proportion of participants reported “often” managing sport-related injuries (37%) and work-related injuries (33%). Patient subgroups most commonly reported as “rarely” or “never” managed included children aged 0–3 years (65%) and patients requiring post-surgical care and rehabilitation (59%).

### Frequency of patient complaints

Supplemental Material 4 describes the frequency with which patient complaints are managed by PBRN participating clinicians from “often” to “never”. Patient complaints which are described as most “often” managed include low back pain without leg pain (96%), neck pain without arm pain (94%), degenerative spine disorders (86%), neck pain with headache (77%), chronic pain (71%) and low back pain with leg pain (71%). Complaints commonly reported as “rarely” or “never” being managed by participating clinicians include non-MSK complaints (70%), wrist and hand pain (60%), ankle and foot pain (45%), elbow pain (41%) and jaw pan (39%).

### Digitalization of chiropractic clinics

More than 50% of PBRN members reported having a fully integrated EHR system within their practice. Of the 44% without an integrated system, 10% reported partial EHR use and 34% reported not using an EHR system. Of clinicians which fully and partially use an EHR system, the most commonly used products were PEX (20%), SiMed (14%) and Chirwin (8%). The remainder of clinicians used a diverse range of products. A larger proportion (85%) of clinicians use encrypted email in their practice. Virtual care or telehealth services are offered by only 5% of participating clinicians. Of those not using telehealth or virtual care, 5% were considering incorporating this service into their practice. Supplementary Material 5 provides further information on digitalization of PBRN participating chiropractic clinics.

### Practice changes due to the COVID-19 pandemic

The majority of PBRN clinicians (68%) reported their quality of life as similar when compared to before the COVID-19 pandemic. Twenty-seven percent reported a worse quality of life, while 5% reported a better quality of life now compared to before the COVID-19 pandemic. Clinicians most often rated their patient numbers and work hours as “unchanged” since the beginning of the COVID-19 pandemic, with 63% and 75% selecting these response options respectively. Supplementary Material 6 provides further information on COVID-19 collected variables.

### Factors associated with motivation to participate in subsequent patient cohort pilot study

A total of 59 participants (39%) rated themselves ≥ 70 on a VAS which asked “On a scale from 0 to 100 how motivated are you to participate in the patient cohort phase of the Swiss ChiCo study”. Multivariable logistic regression showed PBRN members aged between 40 and 59 years were 2.3 times (95% CI 1.0–5.2) more likely to be motivated to participate in the patient cohort study when compared to those aged 39 years or younger. Members within a practice size of 2 or 3 chiropractors were 2.4 times (95% CI 1.1–5.7) and those in a practice size of 4 or more were 2.8 times (95% CI 1.0–7.8) more likely to be motivated to participate in the subsequent study when compared to members engaged in solo practice. No certain evidence of an association was found between the independent variables of sex, language of practice, and EHR use and motivation to participate in the Swiss ChiCo pilot study. Results of the logistic regression analysis are presented in Table 4.

**Table 4:** Logistic regression model for association of age, sex, practice size, language region and EHR use with motivation (≥ 70 on VAS) to participate in subsequent patient cohort pilot study (n = 152).

	OR	95% CI
Age (years)		
≤ 39	Ref	
40–59	2.25	1.01–5.23
≥ 60	3.03	0.85–11.03
Sex		
Male	Ref	
Female	1.62	0.81–3.29
Practice size		
1 chiropractor	Ref	
2 or 3 chiropractors	2.41	1.06–5.68
4 or more chiropractors	2.76	1.01–7.81
Language		
German/Romansh	Ref	
French	0.83	0.35–1.94
Italian	0.96	0.17–4.62
Full EHR use		
Yes	Ref	
No	0.82	0.38–1.79

*CI* confidence intervals, *EHR* electronic health record, *OR* odds ratio, *ref* reference, *y* years.

## Discussion

This paper introduces the Swiss chiropractic PBRN and provides an overview of the demographics and clinical practice characteristics of participating chiropractors in order to encourage nested practice-based research within this infrastructure. The project met pre-specified feasibility objectives of recruitment (approximately 50% of eligible clinicians) and showed an acceptable proportion of clinicians motivated to participate the nested patient cohort study (at least 15 members with a motivation score of ≥ 70). Forty-seven percent of eligible clinicians agreed to participate in the PBRN and 39% of the PBRN was motivated to participate in the nested patient cohort study. Participant clinicians showed high levels of perceived self-confidence in the management low back pain (measured with the PCS) and higher levels of biopsychosocial versus biomedical treatment orientation (measured with the PABS-MSK).

An analysis of Swiss chiropractic PBRN reveals numerous findings of relevance for the chiropractic community and guidance for subsequent projects. Similar to findings in other chiropractic surveys, low back pain and neck pain are the most often managed complaints in chiropractic practices^20^. However, a majority of participant clinicians report “often” managing neck pain with arm pain, low back pain with leg pain, chronic pain conditions, and headaches. The wide range of patient complaints managed by participant clinicians signals an opportunity to conduct research outside of the neck and low back pain paradigm—which is traditionally the case in chiropractic practice-based environments^28–32^. Furthermore, the PBRN provides access to a diverse group of potential patient participants as a majority of participant clinicians reported frequently managing patients 65 years or above, while children aged 4–18 years and ethnic and minority groups were reported as sometimes managed. Access to patient participants, even during the COVID-19 pandemic, appeared viable as most PBRN members described their work hours and patient numbers as unchanged when compared to before. With representation in 110 unique practice locations across Switzerland, data collection from local primary care centres using electronic methods, may allow for an avoidance of public transport, minimization of personal contact, and increased trust in the provision of safety precautions, which have been shown to positively impact research participation during the uncertainty of the pandemic^33^.

With regards to primary clinical and feasibility outcomes, clinicians participating in the PBRN showed high levels of self-confidence for the management of low back pain. These findings are similar to previous work which has shown higher levels of self-confidence in chiropractors compared to primary-care physicians^25^. PBRN clinicians on average scored higher for biopsychosocial versus biomedical treatment orientation on the PABS-MSK. Practitioner treatment orientation has been shown to influence patient management^34,35^. For example, a higher biomedical versus biopsychosocial treatment orientation for low back pain is associated with poor clinical practice guideline adherence and recommendations for delayed return to work and activity^34,36^. Both the PCS and the PABS have been used as relevant outcome measures for the assessment of confidence and treatment perceptions after practitioner training interventions^37,38^.

The Swiss chiropractic PBRN was proportionally more female and slightly younger than the larger Swiss chiropractic community. Of the participating clinicians, females and members practicing in large clinics were more likely to be motivated to participate in the patient cohort study. Clinicians who participate in voluntary research have been shown to have a different demographic and clinical profile compared to those who do not^39^. Generally, our findings are similar to previous published work which show higher voluntary research participation in clinicians who are younger, female, more engaged in-patient care and practice in non-solo clinics^39–41^. Older clinicians within the PBRN were more likely to express motivation to participate in the patient cohort pilot study when compared to younger clinicians. It can be hypothesized that factors such as increased comfort with clinical routine, well-established patient relationships, practice ownership, and a larger number of rostered patients may have led to increased motivation to support patient-level data collection.

The Swiss Chiropractic PBRN has been designed using a sub-study PBRN model^13^. Typically, this method first establishes a practitioner database through self-report questionnaires, which provides an initial framework to conduct subsequent projects nested within the PBRN infrastructure. Successfully implemented PBRNs developed using a similar model include ACORN and the Osteopathic PBRNs of Australia (Osteopathic research innovation network (ORION)) and New Zealand (Osteopathic research connect—New Zealand (ORC-NZ))^14,42^. All 3 networks have recruited a substantial proportion of clinician participants and have conducted substudies—the majority of which are cross-sectional and further describe the clinician population and practice characteristics. In contrast, the first nested project within the Swiss Chiropractic PBRN (the Swiss ChiCo pilot study) is registered as a 12-week patient prospective cohort study to describe the clinical course of chiropractic patients with new-conservative healthcare seeking for MSK-based pain. Perhaps more notably the Swiss ChiCo pilot study will assess the feasibility of collecting patient-level information longitudinally within a sub study PBRN model. This method of collecting data is more commonly seen in clinical registers, which use centralized coordinated record keeping systems^43,44^. Establishing feasibility for patient-level longitudinal data capture is of importance as it garners confidence amongst stakeholders and external researchers for conducting a wide range of subsequent research within the Swiss chiropractic PBRN infrastructure.

Our study has several limitations. First, we only used an electronic data collection approach which may have led to selective participation of clinicians with higher levels of digital literacy. Second, this Swiss Chiropractic PBRN study collected information through self-report and was subject to recall bias. Data quality may have been improved by asking clinicians to perform a chart review prior to completing the PBRN entry questionnaire. However, further understanding of specific patient complaints may be better described with subsequent nested research within the PBRN. Third, ongoing maintenance and expansion of the PBRN is highly dependent on continued stakeholder engagement and support.

## Conclusion

The Swiss chiropractic PBRN recruited approximately half of Swiss chiropractic clinicians in over one-hundred unique clinical practices across Switzerland. The PBRN is largely representative when comparted to the larger Swiss chiropractic population with regards to age, language, and location. The flexible nature of the PBRN allows for continued recruitment and the formulation of diverse types of nested research.

## Supplementary Information

Supplementary Information.

## Data Availability

Data from the Swiss chiropractic PBRN will be made available for research purposes. Requests, including a synopsis of the study plan, can be addressed to the corresponding author.
