# Supplementary material for: The Swiss chiropractic practice-based research network: a population-based cross-sectional study to inform future musculoskeletal research
Source: Sci Rep. 2023 Apr 6;13:5655. doi: 10.1038/s41598-023-32437-3 (PMC10078089; doi:10.1038/s41598-023-32437-3)
Supplement: Supplementary file 1 — Supplementary Information. [file 41598_2023_32437_MOESM1_ESM.pdf]

The Swiss chiropractic practice-based research network: a population-based cross-sectional study to inform future musculoskeletal research

Rahim Lalji DC MSc[1,2,3], Léonie Hofstetter DCM[1], Alice Kongsted PhD[4,5], Viktor von Wyl PhD[2,6], Milo A Puhan MD PhD[2], Cesar A Hincapié DC PhD[1,2,3]

[1] EBPI-UWZH Musculoskeletal Epidemiology Research Group, University of Zurich and Balgrist University Hospital, Zurich, Switzerland

[2] Epidemiology, Biostatistics and Prevention Institute (EBPI), University of Zurich, Zurich, Switzerland

[3] University Spine Centre Zurich (UWZH), Balgrist University Hospital, University of Zurich, Zurich, Switzerland

[4] Department of Sports Science and Clinical Biomechanics, University of Southern Denmark, Odense, Denmark

[5] Chiropractic Knowledge Hub, Odense, Denmark

[6] Institute for Implementation Science in Health Care, University of Zurich, Zurich, Switzerland

TABLE OF CONTENTS

SUPPLEMENTAL MATERIAL 1: STROBE checklist for cross-sectional studies...................... 1

SUPPLEMENTAL MATERIAL 2: PBRN entry questionnaire 5

SUPPLEMENTAL MATERIAL 3: Frequency of patient subgroups managed........................... 12

SUPPLEMENTAL MATERIAL 4: Frequency of patient subgroups managed........................... 13

SUPPLEMENTAL MATERIAL 5: Digitalization of PBRN participating practices 14

SUPPLEMENTAL MATERIAL 6: Practice changes due to the COVID-19 pandemic............. 15

SUPPLEMENTAL MATERIAL 1: STROBE checklist for cross-sectional studies

Topic	Item No	Recommendation
**Title and abstract**	1	(*a*) Indicate the study's design with a commonly used term in the title or the abstract<br>"The Swiss chiropractic practice-based research network: a population based cross-sectional study to inform future musculoskeletal research"
		(*b*) Provide in the abstract an informative and balanced summary of what was done and what was found<br>"A population-based cross-sectional study was performed. PBRN clinician characteristics were described and factors related to motivation (operationalized as VAS score ≥70) to participate in a subsequent patient cohort pilot study were assessed."<br><br>"Among 326 eligible chiropractors, 152 enrolled in the PBRN (47% participation). The PBRN was representative of the larger Swiss chiropractic population with regards to age, language, and geographic distribution."
**Introduction**		
Background/rationale	2	Explain the scientific background and rationale for the investigation being reported<br>"Musculoskeletal (MSK) pain conditions, such as neck pain and low back pain, are a leading cause of disability globally and are the most prevalent disease area which would benefit from rehabilitation."<br><br>"As a large proportion of MSK pain is managed in primary care, efforts to improve the quality of care in these settings, such as the development of practice-based research networks (PBRNs), may play an important role in identifying, studying, and addressing similar practice-based gaps."
Objectives	3	State specific objectives, including any prespecified hypotheses<br>"The overarching aim of this study is to describe the characteristics of chiropractors recruited to a newly developed Swiss chiropractic PBRN and the representativeness of the PBRN in comparison to the larger Swiss clinician population to facilitate subsequent collaborative practice-based research."
**Methods**		
Study design	4	Present key elements of study design early in the paper<br>"We reported this population-based cross-sectional study according to the Strengthening the Reporting of Observational Studies in Epidemiology (STROBE for cross-sectional studies) statement."
Setting	5	Describe the setting, locations, and relevant dates, including periods of recruitment, exposure, follow-up, and data collection<br>"All 326 registered active chiropractor members (fully licensed chiropractors and postgraduate assistant chiropractors) of ChiroSuisse were eligible and invited to participate in the PBRN. This included members with clinical practice locations in Switzerland and Liechtenstein."<br><br>"From September 9th 2021 to December 19th 2021, clinicians were provided the opportunity to sign up for the Swiss chiropractic PBRN through scanning a Quick Response (QR) code at an in-person ChiroSuisse event or through a web link via email invitation."
Participants	6	(*a*) Give the eligibility criteria, and the sources and methods of selection of participants<br>"All 326 registered active chiropractor members (fully licensed chiropractors and postgraduate assistant chiropractors) of ChiroSuisse were eligible and invited to participate in the PBRN. This included members with clinical practice locations in Switzerland and Liechtenstein."
Variables	7	Clearly define all outcomes, exposures, predictors, potential confounders, and effect modifiers. Give diagnostic criteria, if applicable<br>"Primary clinical outcome of perceived self-confidence for the management of low back pain was measured using the Practitioner Confidence Scale ((PCS) range 4-20, lower scores mean greater self-confidence) and biomedical versus biopsychosocial treatment orientation was measured using the MSK version of the Pain Attitudes and Beliefs Scale (PABS-MSK, range 10-60 each, with higher scores meaning greater treatment orientation)."<br><br>"The primary feasibility outcome of motivation to participate in the nested Swiss ChiCo patient cohort pilot study was measured using a Visual Analog Scale ((VAS), range 0-100), higher scores indicate greater motivation)."

Topic	Item No	Recommendation
Data sources/ measurement	8*	For each variable of interest, give sources of data and details of methods of assessment (measurement). Describe comparability of assessment methods if there is more than one group<br>"Primary clinical outcome of perceived self-confidence for the management of low back pain was measured using the Practitioner Confidence Scale ((PCS) range 4-20, lower scores mean greater self-confidence) and biomedical versus biopsychosocial treatment orientation was measured using the MSK version of the Pain Attitudes and Beliefs Scale (PABS-MSK, range 10-60 each, with higher scores meaning greater treatment orientation)."<br><br>"The primary feasibility outcome of motivation to participate in the nested Swiss ChiCo patient cohort pilot study was measured using a Visual Analog Scale ((VAS), range 0-100), higher scores indicate greater motivation). For this question, participants were asked "On a scale from 0-100 how motivated are you to participate in the patient cohort phase of the Swiss ChiCo pilot study".
Bias	9	Describe any efforts to address potential sources of bias<br>"Clinicians were provided the opportunity to sign up for the Swiss chiropractic PBRN through scanning a Quick Response (QR) code at in-person ChiroSuisse event or through a web link via email invitation."<br><br>"Before full implementation, the PBRN entrance questionnaire was pilot tested by licenced chiropractors from all Swiss national language regions (German, French and Italian)."
Study size	10	Explain how the study size was arrived at<br>"Approximately 98% of all chiropractors in Switzerland are members of ChiroSuisse (personal communication, April 22, 2021), which corresponded to 326 clinician members as of December 2021"
Quantitative variables	11	Explain how quantitative variables were handled in the analyses. If applicable, describe which groupings were chosen and why<br>"Independent variables included in the regression model were selected on the basis of clinical experience and prior analysis of factors related to EHR use for Swiss chiropractors."
Statistical methods	12	(*a*) Describe all statistical methods, including those used to control for confounding<br>"Data were extracted from REDCap into R (version 4.2.0) for analysis. Descriptive statistics were reported as raw numbers with percentages or means with standard deviations as appropriate. Primary clinical and feasibility outcomes were additionally described with 95% CIs for mean values and percentages. Multivariable logistic regression was used to assess the association between clinician and practice characteristics (age, sex, practice size, language of practice, EHR use) and motivation to participate in the patient cohort pilot study (Yes/No, cut point operationalised as VAS score ≥70). Alpha level was set at 0.05 and results were reported as odds ratios (ORs) with 95% CIs."
		(*b*) Describe any methods used to examine subgroups and interactions<br>N/A
		(*c*) Explain how missing data were addressed<br>"Only clinicians who completed the electronic informed consent and fully completed the entry questionnaire were considered as part of the PBRN and available for future nested study recruitment."
		(*d*) If applicable, describe analytical methods taking account of sampling strategy<br>N/A
		(*e*) Describe any sensitivity analyses<br>N/A
**Results**		
Participants	13*	(a) Report numbers of individuals at each stage of study—eg numbers potentially eligible, examined for eligibility, confirmed eligible, included in the study, completing follow-up, and analysed<br>"Of the 326 eligible chiropractors, 152 (46.6%) agreed to participate and completed the PBRN entrance questionnaire, 24 declined to participate and 150 did not respond (174 nonparticipants).
		(b) Give reasons for non-participation at each stage<br>Of those who declined, lack of time was reported as the most common reason for non-participation (50%).
		(c) Consider use of a flow diagram<br>Flow diagram presented as Fig. 1

Topic	Item No	Recommendation
Descriptive data	14*	(a) Give characteristics of study participants (eg demographic, clinical, social) and information on exposures and potential confounders<br>"Clinician participants of this Swiss chiropractic PBRN enrollment cross-sectional study were generally balanced in terms of sex (53% male versus 47% female) with an average age of 47 years (SD=12.4). The majority were fully licenced chiropractors (87%), with the remainder being postgraduate chiropractors (13%)"<br><br>"The most common language participating clinicians use in their practice was reported as German (69%), followed by French (25%), Italian (5%) and finally Romansh (1%)."
		(b) Indicate number of participants with missing data for each variable of interest<br>"Only clinicians who completed the electronic informed consent and fully completed the entry questionnaire were considered as part of the PBRN and available for future nested study recruitment."
Outcome data	15*	Report numbers of outcome events or summary measures<br>Of the 326 eligible chiropractors, 152 (46.6%) agreed to participate and completed the PBRN entrance questionnaire.<br><br>On average PBRN participating clinicians showed high confidence for managing patients with low back pain (5.6, SD = 1.8; 95%CI 5.3 to 5.9). The mean score on the biomedical subscale of the PABS-MSK was 32 (SD = 6.8; 95%CI 31.4 to 33.5), while mean score on the biopsychosocial subscale was 52 (SD = 5.0; 95%CI 50.9 to 52.5). 39% of participating clinicians were motivated (VAS score ≥ 70) to participate in the Swiss ChiCo pilot nested PBRN study (95%CI = 30% to 46%) and average motivation to participate was 50 (SD = 32.3; 95%CI = 45.0 to 55.6).
Main results	16	(*a*) Give unadjusted estimates and, if applicable, confounder-adjusted estimates and their precision (eg, 95% confidence interval). Make clear which confounders were adjusted for and why they were included<br>"Independent variables included in the regression model were selected on the basis of clinical experience and prior analysis of factors related to EHR use for Swiss chiropractors."
		(*b*) Report category boundaries when continuous variables were categorized<br>"A pragmatic decision was made a-priori to identify clinicians who reported a well above medium interest to participate in the Swiss ChiCo pilot study based on a VAS score of ≥70 (hereafter denoted as "motivated PBRN participants".
		(*c*) If relevant, consider translating estimates of relative risk into absolute risk for a meaningful time period<br>N/A
Other analyses	17	Report other analyses done—eg analyses of subgroups and interactions, and sensitivity analyses<br>N/A
**Discussion**		
Key results	18	Summarise key results with reference to study objectives<br>"The project met pre-specified feasibility objectives of recruitment (approximately 50% of eligible clinicians) and showed an acceptable proportion of clinicians motivated to participate the nested patient cohort study (at least 15 members with a motivation score of ≥70). Forty-seven percent of eligible clinicians agreed to participate in the PBRN and 39% of the PBRN was motivated to participate in the nested patient cohort study. Participant clinicians showed high levels of perceived self-confidence in the management low back pain (measured with the PCS) and higher levels of biopsychosocial versus biomedical treatment orientation (measured with the PABS-MSK)."
Limitations	19	Discuss limitations of the study, taking into account sources of potential bias or imprecision. Discuss both direction and magnitude of any potential bias<br>"Our study has several limitations. First, we only used an electronic data collection approach which may have led to selective participation of clinicians with higher levels of digital literacy."<br><br>"Second, this Swiss Chiropractic PBRN study collected information through self-report and was subject to recall bias. Data quality may have been improved by asking clinicians to perform a chart review prior to completing the PBRN entry questionnaire."
Interpretation	20	Give a cautious overall interpretation of results considering objectives, limitations, multiplicity of analyses, results from similar studies, and other relevant evidence<br>"The Swiss chiropractic PBRN recruited approximately half of Swiss chiropractic clinicians in over one-hundred unique clinical practices across Switzerland. The PBRN is largely representative when comparted to the larger Swiss chiropractic population with regards to age, language, and location."

Topic	Item No	Recommendation
Generalisability	21	Discuss the generalisability (external validity) of the study results<br>"The Swiss chiropractic PBRN was proportionally more female and slightly younger than the larger Swiss chiropractic community. Of the participating clinicians, females and members practicing in large clinics were more likely to be motivated to participate in the patient cohort study"
**Other information**		
Funding	22	Give the source of funding and the role of the funders for the present study and, if applicable, for the original study on which the present article is based<br>"This work was internally supported at the University of Zurich through funding from the Foundation for the Education of Chiropractors in Switzerland. The funder had no role in the study conceptualization and design, or in the collection, analysis, interpretation of data, writing of the report or decision to submit the manuscript for publication."

SUPPLEMENTAL MATERIAL 2: PBRN entry questionnaire

Dr. [name_last], thank you for your participation in this study.

Questions on this survey will ask about clinician demographics, practice characteristics, clinician confidence in the management of low back pack pain, practitioner attitudes and beliefs towards MSK complaints, digitalization of chiropractic practices with regards to EPR and encrypted email use, and how COVID-19 has changed clinical practice.

All information is collected securely and kept confidential.

1. Clinic name ___________________________________ (Your clinic's name)

2. Clinic address:
   Street name and number ___________________________________
   PLZ / NPA ___________________________________
   City / Town ___________________________________

3. Sex
   - Male
   - Female

4. ChiroSuisse member classification
   - Assistant / Resident, first year
   - Assistant / Resident, second year
   - Fully licensed chiropractor

5. Years of chiropractic practice ___________________________________ (Number of years practicing as a chiropractor)

6. Average number of patients seen per week over the last 3 months
   - < 50
   - 50-99
   - 100-149
   - 150-199
   - 200-249
   - ≥ 250

7. Average number of new patients seen per week over the last 3 months ("New" is defined as not having visited you before for a MSK complaint)
   - 0
   - 1-3
   - 4-6
   - 7-9
   - 10-12
   - 13-15
   - 16-20
   - 20

8. How many chiropractors work at your clinic? (Number of chiropractors including yourself)

- 1
- 2
- 3
- 4
- 5
- 6 or more

9. Do you work with other healthcare professionals besides chiropractors?
- Yes
- No

9a (If [Yes] to 9). How many other healthcare professionals work at your clinic?
- 1
- 2
- 3
- 4
- 5
- 6 or more

9b. (If [Yes] to 9). Other healthcare practitioners involved in the practice (please select all that apply)?
- Physiotherapist
- Massage therapist
- Medical doctor
- Acupuncturist
- Nutritionist
- Other ______

10. What language do you primarily use in your practice?
- Deutsch
- Français
- Italiano
- Romansh
- English
- Other ______

Types of patients and patient complaints seen within practice

Dr. [name_last], from "often" to "never" please select the best option as it relates to the frequency of clinical complaints and the types of patients you see in practice

11. Frequency with each condition is managed in your practice				
	Often	Sometimes	Rarely	Never
Neck pain without arm pain				
Neck pain with arm pain				
Neck pain with headache				
Thoracic spine and rib pain				
Low back pain without leg pain				
Low back pain with leg pain				
Shoulder pain				

Elbow pain				
Wrist and hand pain				
Hip pain				
Knee pain				
Ankle and foot pain				
Jaw pain / TMJ pain				
Degenerative spine disorders				
Other degenerative joint disorders				
Postural disorders				
Headaches				
Tendinopathy				
Chronic pain				
Spinal health maintenance				
Non MSK complaints				

12. Frequency with which each patient type is managed in your practice				
Children (0-3 years of age)	Often	Sometimes	Rarely	Never
Children (4-18 years of age)				
Older persons (≥ 65 years of age)				
Pregnant women				
Motor-vehicular accident injuries				
Work-related injuries				
Sport-related injuries				
Post-surgical care and rehabilitation				
Ethnic and minority groups				

Practitioner Confidence Scale

Dr. [name_last], the survey below is called the Practitioner Confidence Scale. It has been developed to measure a clinician's confidence in managing people with low back pain.

1. I lack the diagnostic tools or knowledge needed to effectively assess patients with low back pain
    - Strongly agree
    - Agree
    - Not sure
    - Disagree
    - Strongly disagree

2. I know exactly what to do to effectively treat patients with low back pain
    - Strongly agree
    - Agree
    - Not sure
    - Disagree
    - Strongly disagree

3. I am very comfortable treating patients with low back pain

- Strongly agree
- Agree
- Not sure
- Disagree
- Strongly disagree

4. How well prepared to manage low back pain are you?
   - Very well
   - Well
   - Adequately
   - Poorly
   - Very poorly

5 I feel confident using psychological and behavioural elements in the treatment of low back pain patients
- Strongly agree
- Agree
- Not Sure
- Disagree
- Strongly Disagree

6. I feel confident working with a patient with low back pain not basing this on a structural diagnosis
   - Strongly agree
   - Agree
   - Not sure
   - Disagree
   - Strongly disagree

Pain attitudes and beliefs scale

Dr. [name_last], we are interested in your views about non-specific musculoskeletal pain. Please indicate the extent to which you agree or disagree with the following statements about non-specific musculoskeletal pain by putting a tick in one box on each line. Please provide an answer to all the questions. If a question does not seem directly relevant to your clinical practice, please try to provide an answer based on your belief or instinct, rather than leave it blank

	Totally disagree	Largely disagree	Disagree to some extent	Agree to some extent	Largely agree	Totally agree
A patient's beliefs about the cause of their musculoskeletal pain must be understood						
If musculoskeletal pain increases in severity, I immediately adjust the intensity of treatment accordingly						
Increased pain indicates new tissue damage or the spread of existing damage						
Specific and realistic goals for treatment must be agreed						

The reaction of a patient's family and friends will promote recovery						
If therapy does not result in a reduction in musculoskeletal pain, there is a high risk of severe restrictions in the long term						
Patients with musculoskeletal pain should preferably practice only pain free movements						
Reducing a patient's fear is essential to the treatment process						
If patients complain of pain during exercise, I worry that damage is being caused						
A patient's perceived barriers to work must be assessed						
The severity of tissue damage determines the level of pain						
A patient's physical activity level should be considered in the management of their musculoskeletal pain problem						
How a patient currently copes with their pain must be assessed						
A patient's expectations about treatment for musculoskeletal pain affect their outcome						
In the long run, patients with musculoskeletal pain have a higher risk of developing functional impairments						
Pain reduction is a precondition for the restoration of normal functioning						
Pain is a nociceptive stimulus, indicating tissue damage						
Musculoskeletal pain indicates the presence of organic injury						
Biological, psychological and social factors should be included in the clinical assessment						
I consider a patient's social support network in my clinical management						

Digitalization of chiropractic clinics

Dr. [name_last], the following questions relate to your use of electronic patient records and encrypted email use in practice

1. Do you use an electronic patient record (EPR) system for clinical record keeping in your practice?
   - Yes. I use only an EPR system for clinical record keeping in practice
   - Partially. I use a mix of an EPR and paper-based system for clinical record keeping in practice
   - No. I use a paper-based system currently, but am considering switching to an EPR system

- No. I use only a paper-based system for clinical record keeping in practice.

1a. (If [Yes or Partially] to 1). Please indicate the Manufacturer Name and Product Name for the EPR information system that you use in practice. (Please enter both the EPR Manufacturer and Product names)

____

1b. (If [No but considering] to 1). Please indicate the Manufacturer Name and Product Name for the EPR information system that you are considering to use in practice. (Please enter both the EPR Manufacturer and Product names)

____

2. Do you use a secure/encrypted email system for patient communication in your practice (e.g., HIN or ProtonMail)
   - Yes
   - No

2b. (If [Yes] to 1). Please indicate the Product Name for the secure/encrypted email system you use in practice. (Please enter the secure/encrypted email system Product name)

____

How COVID-19 has changed clinical practice

Dr. [name_last], the following questions relate to how the COVID-19 pandemic has affected you and your chiropractic practice.

1. How would you compare your quality of life now, when compared to before the COVID-19 pandemic?
   - Better
   - Similar
   - Worsened

2. How have your patient numbers been affected since the start of the COVID-19 pandemic?
   - Increased
   - Unchanged
   - Decreased

3. Have you changed your work hours since the start of the COVID-19 pandemic?
   - Increased
   - Unchanged
   - Decreased

4. Does your clinic offer telehealth/virtual care services?
   - Yes
   - No
   - No, but I am considering integrating it into my practice

5. (If [Yes] to 1). How has patient use of telehealth or virtual care services changed since the start of the COVID-19 pandemic?

- Increased use
- Unchanged
- Decreased use

Motivation for cohort study involvement

Dr. [name_last], a small number of clinics within this newly developed research network will be contacted to participate in the recruitment of patient participants for the patient cohort phase of the Swiss ChiCo study.

This will involve:

1) Providing new care seeking patients with a study flyer and study tablet to judge their level of interest in participating in this study; 2) asking patients consecutively whether they would be interested in participating; 3) allowing patients time to read the study information sheet and perform electronic informed consent on the study tablet

1) On a scale from 0 - 100 how motivated are you to participate in the patient cohort phase of the Swiss ChiCo study?

0 (not motivated at all) 100 (highly motivated)

(Place a mark on the scale above)

SUPPLEMENTAL MATERIAL 3: Frequency of patient subgroups managed. Abbreviations: y, years. Values which are not provided represent less than 5% of responses.

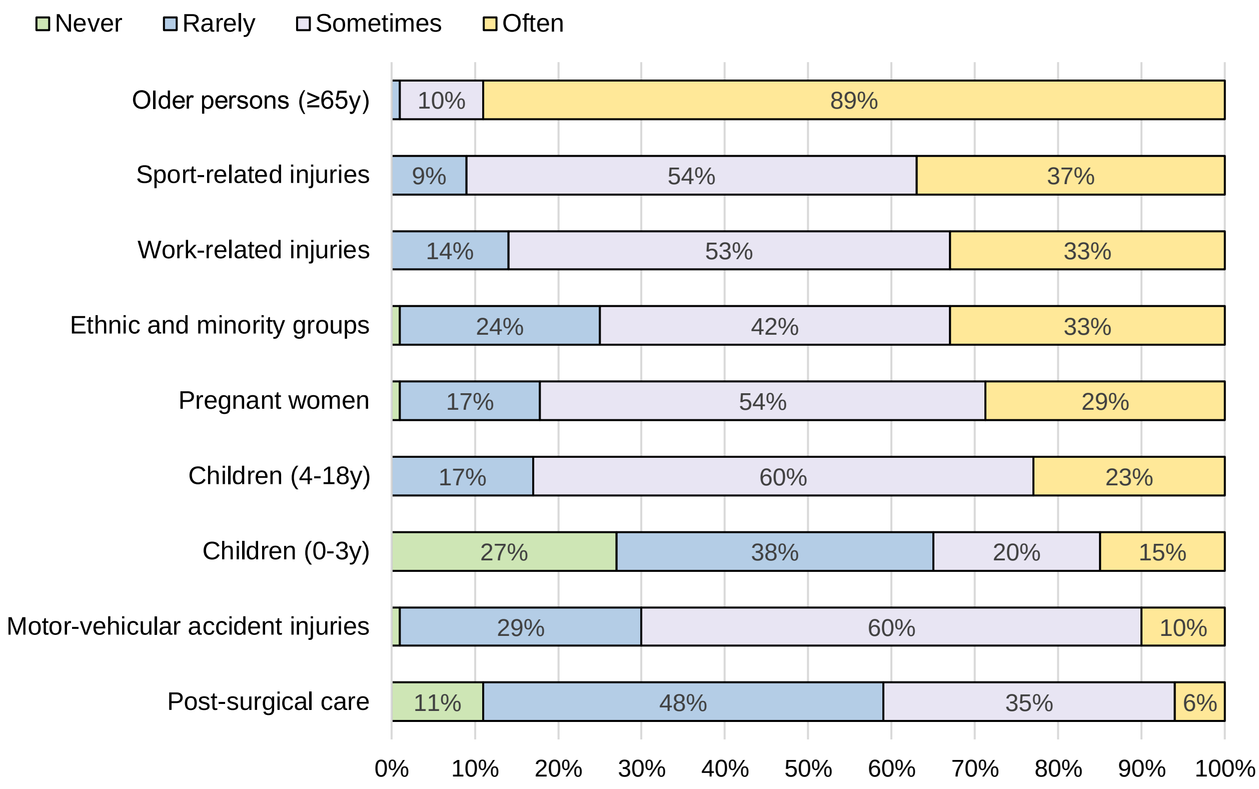

SUPPLEMENTAL MATERIAL 4: Frequency of patient subgroups managed. Abbreviations: MSK, musculoskeletal. Values which are not provided represent less than 5% of responses.

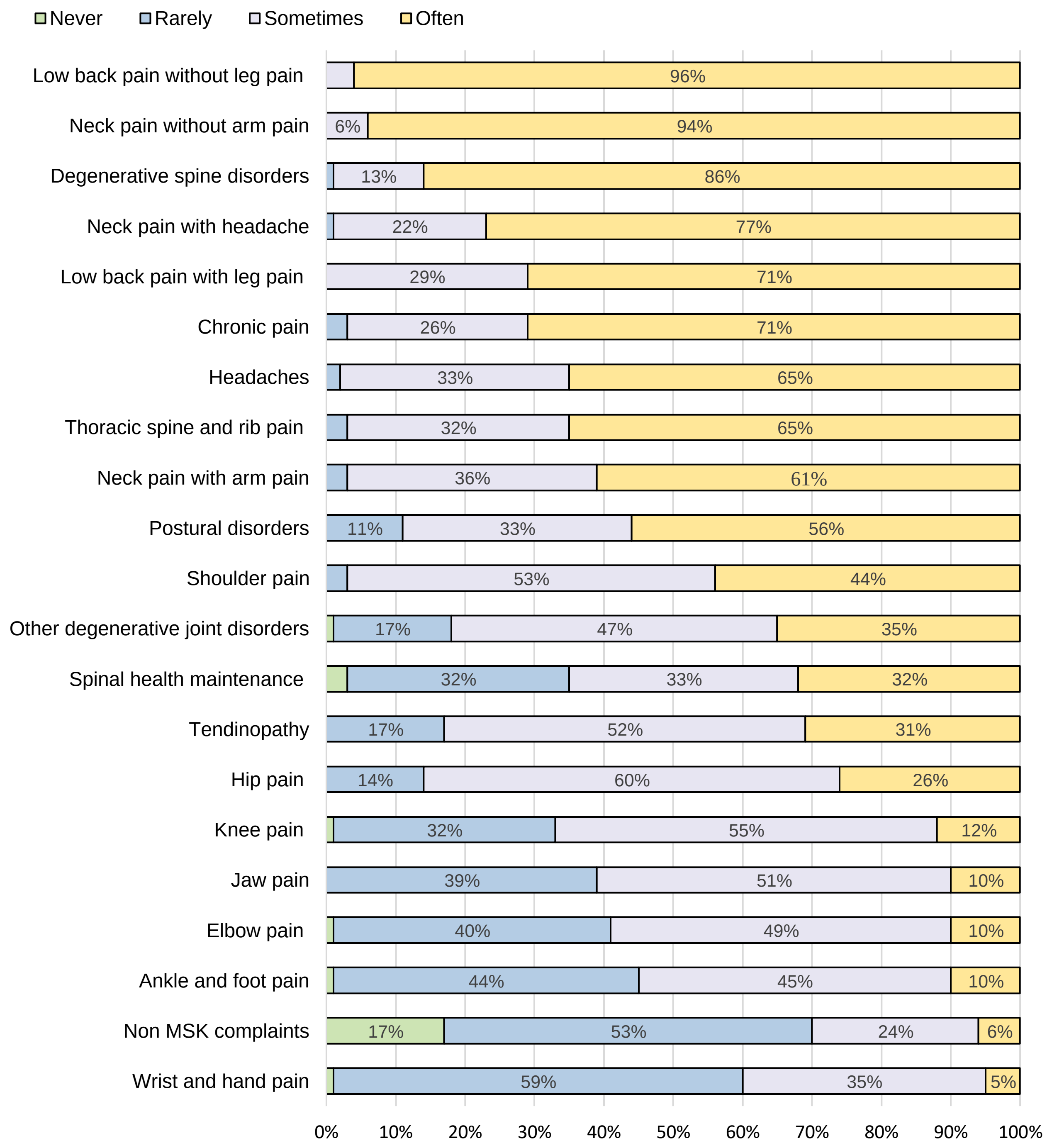

SUPPLEMENTAL MATERIAL 5: Digitalization of PBRN participating practices.
Abbreviations: CI, confidence intervals; EHR, electronic health record; n, number

Digitalization in practice	PBRN participants (n = 152)	95% CI	Motivated participants (n = 59)	95% CI
**EHR use in practice**				
Yes	85 (56%)	48 to 64%	35 (59%)	47 to 71%
Partial use	15 (10%)	6 to 16%	8 (14%)	7 to 25%
No	52 (34%)	27 to 42%	16 (27%)	17 to 40%
**Most common used EHR systems (participants, n = 100; motivated, n = 43)**				
PEX	20 (20%)	13 to 29%	7 (16%)	8 to 30%
siMed	14 (14%)	9 to 22%	6 (14%)	7 to 27%
Chirwin	8 (8%)	4 to 15%	3 (7%)	2 to 19%
VitoData	7 (7%)	3 to 14%	5 (12%)	5 to 24%
Curamed	5 (5%)	2 to 11%	2 (4%)	1 to 15%
KISIM	5 (5%)	2 to 11%	5 (12%)	5 to 24%
Other	41 (41%)	32 to 51%	15 (35%)	22 to 50%
**Encrypted email use in practice**				
Yes	129 (85%)	78 to 90%	54 (92%)	82 to 96%
No	23 (15%)	10 to 22%	5 (8%)	4 to 18%
**Virtual care/telehealth services**				
Yes	8 (5%)	3 to 10%	5 (8%)	4 to 18%
No, but considering	8 (5%)	3 to 10%	3 (5%)	2 to 14%
No	136 (90%)	84 to 93%	51 (87%)	75 to 93%

SUPPLEMENTAL MATERIAL 6: Practice changes due to the COVID-19 pandemic:
Abbreviations: CI, confidence Intervals; n, number; QOL, quality of life

COVID-19 response options	PBRN participants (n = 152)	95%CI	Motivated Participants (n = 59)	95%CI
**QOL now compared to start of the COVID-19 pandemic**				
Better	7 (5%)	2 to 9%	1 (2%)	0 to 9%
Similar	103 (68%)	60 to 75%	42 (71%)	59 to 81%
Worsened	42 (27%)	21 to 35%	16 (27%)	17 to 40%
**Change in patient numbers compared to start of COVID-19 pandemic**				
Increased	20 (13%)	9 to 19%	10 (17%)	9 to 28%
Unchanged	96 (63%)	55 to 70%	36 (61%)	48 to 72%
Decreased	36 (24%)	18 to 31%	13 (22%)	13 to 34%
**Change in work hours compared to start of COVID-19 pandemic**				
Increased	18 (12%)	8 to 18%	9 (15%)	8 to 27%
Unchanged	115 (75%)	68 to 82%	44 (75%)	62 to 84%
Decreased	19 (13%)	8 to 19%	6 (10%)	5 to 20%
